# 1,5-Anhydro-2-de­oxy-1,2-*C*-dichloro­methyl­ene-3,4,6-tri-*O*-(4-meth­oxy­benz­yl)-d-*glycero*-d-*gulo*-hexitol

**DOI:** 10.1107/S1600536811026870

**Published:** 2011-07-13

**Authors:** Henok H. Kinfe, Paseka T. Moshapo, Alfred Muller

**Affiliations:** a Research Center in Synthesis and Catalysis, Department of Chemistry, University of Johannesburg (APK Campus), PO Box 524, Auckland Park, Johannesburg 2006, South Africa

## Abstract

The pyranosyl ring in the title compound, C_31_H_34_Cl_2_O_7_, adopts a twist-boat conformation. The 4-meth­oxy­benzyl groups are located in equatorial positions with the meth­oxy groups nearly coplanar with their respective rings [dihedral angles of 0.2 (3) and 9.4 (2)°]. The aromatic rings adopt orientations enabling them to participate in C—H⋯π inter­actions with neighboring meth­oxy groups. The crystal structure is additionally stabilized by weak C—H⋯O inter­actions.

## Related literature

For the synthesis and chemistry of cyclo­propanated carbohydrates, see: Cousins & Hoberg (2000 ▶); Yu & Pagenkopf (2005 ▶). For the modified Simmons–Smith reaction route of preparing cyclo­propanated sugars, see: Gammon *et al.* (2007 ▶); Ramana *et al.* (1997 ▶); Murali *et al.* (1995 ▶); Boeckman *et al.* (1987 ▶); Hoberg & Bozell (1995 ▶). For the dihalocarbene cyclo­propanation route, see: Gammon *et al.* (2007 ▶); Ramana *et al.* (1997 ▶); Murali *et al.* (1995 ▶); Brimacombe *et al.* (1967 ▶); Weber & Hall (1979 ▶). For the diazo­cyclo­propanation route, see: Hoberg & Claffey (1996 ▶); Henry & Fraser-Reid (1995 ▶); Timmers *et al.* (1996) ▶. For ring puckering analysis, see: Cremer & Pople (1975 ▶). For standard bond lengths, see: Allen *et al.* (1987 ▶).
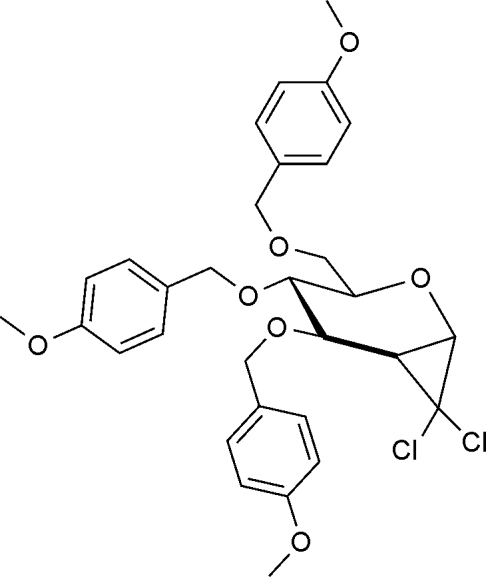


## Experimental

### 

#### Crystal data

C_31_H_34_Cl_2_O_7_
*M* *_r_* = 589.48
Monoclinic, 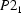

*a* = 5.3480 (4) Å
*b* = 18.1110 (14) Å
*c* = 14.8230 (11) Å
β = 91.162 (2)°
*V* = 1435.43 (19) Å^3^
*Z* = 2
Mo *K*α radiation
μ = 0.27 mm^−1^
*T* = 100 K
0.53 × 0.44 × 0.39 mm

#### Data collection

Bruker KappaCCD APEX DUO 4K diffractometer
Absorption correction: multi-scan (*SADABS*; Bruker, 2007 ▶) *T*_min_ = 0.869, *T*_max_ = 0.901
13864 measured reflections
4689 independent reflections
4635 reflections with *I* > 2σ(*I*)
*R*_int_ = 0.026

#### Refinement

*R*[*F* ^2^ > 2σ(*F* ^2^)] = 0.023
*wR*(*F* ^2^) = 0.062
*S* = 1.04
4689 reflections
364 parameters
1 restraint
H-atom parameters constrained
Δρ_max_ = 0.28 e Å^−3^
Δρ_min_ = −0.16 e Å^−3^
Absolute structure: Flack (1983 ▶), 1023 Friedel pairs
Flack parameter: 0.03 (3)

### 

Data collection: *APEX2* (Bruker, 2007 ▶); cell refinement: *SAINT-Plus* (Bruker, 2007 ▶); data reduction: *SAINT-Plus* and *XPREP* (Bruker, 2007 ▶); program(s) used to solve structure: *SIR97* (Altomare *et al.*, 1999 ▶); program(s) used to refine structure: *SHELXL97* (Sheldrick, 2008) ▶; molecular graphics: *DIAMOND* (Brandenburg & Putz, 2005 ▶); software used to prepare material for publication: *WinGX* (Farrugia, 1999 ▶).

## Supplementary Material

Crystal structure: contains datablock(s) global, I. DOI: 10.1107/S1600536811026870/mw2012sup1.cif

Structure factors: contains datablock(s) I. DOI: 10.1107/S1600536811026870/mw2012Isup2.hkl

Additional supplementary materials:  crystallographic information; 3D view; checkCIF report

## Figures and Tables

**Table 1:** Hydrogen-bond geometry (Å, °) *Cg*1, *Cg*2 and *Cg*3 are the centroids of the C17–C22, C9–C14 and C25–C30 rings, respectively.

*D*—H⋯*A*	*D*—H	H⋯*A*	*D*⋯*A*	*D*—H⋯*A*
C4—H4⋯O1^i^	1.00	2.38	3.3487 (16)	164
C23—H23*B*⋯O7^ii^	0.98	2.50	3.399 (2)	153
C26—H26⋯O3^i^	0.95	2.54	3.2301 (16)	130
C31—H31*C*⋯O1^iii^	0.98	2.49	3.466 (2)	172
C15—H15*A*⋯*Cg*1^iv^	0.98	2.95	3.927 (3)	174
C15—H15*C*⋯*Cg*2^v^	0.98	2.99	3.873 (2)	151
C24—H24*B*⋯*Cg*3^v^	0.99	2.90	3.7983 (17)	152

Symmetry codes: (i) 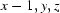; (ii) 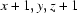; (iii) 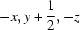; (iv) 
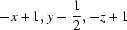
; (v) 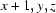.

**Fig. 1.**
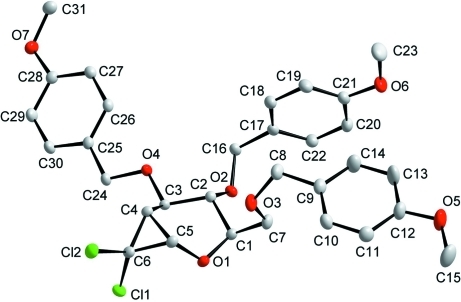
View of I. Displacement ellipsoids are drawn at a 50% probability level. Hydrogen atoms are omitted for clarity.

**Fig. 2.**
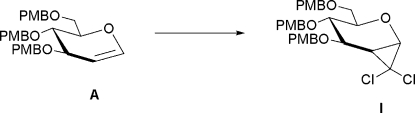
Reaction scheme for the dihalocarbene cyclopropanation of the protected glucal (PMB = p-methoxybenzyl).

## supplementary crystallographic information

### Comment

1,2-Cyclopropanated sugars have found widespread applications in organic synthesis (Cousins & Hoberg, 2000; Yu & Pagenkopf, 2005). The high reactivity of strained cyclopropanes in conjunction with the inherent optical purity of sugars makes cyclopropanated carbohydrates indispensable chiral building blocks. Due to the resemblance of cyclopropyl to an olefinic functionality and assistance from the lone pair of electrons on the pyran ring oxygen atom, 1,2-cyclopropanated sugars undergo regioselective ring opening to afford 2-C branched and C-1 functionalized sugar derivatives (Gammon *et al.*, 2007; Ramana *et al.*, 1997; Murali *et al.*, 1995). The most common methods of preparing cyclopropanated sugars are: the modified Simmons-Smith reaction (see Gammon *et al.*, 2007; Ramana *et al.*, 1997; Murali *et al.*, 1995; Boeckman *et al.*, 1987; Hoberg & Bozell, 1995), dihalocarbene cyclopropanation (see Gammon *et al.*, 2007; Ramana *et al.*, 1997; Murali *et al.*, 1995; Brimacombe *et al.*, 1967; Weber & Hall, 1979) and diazocyclopropanation (see Hoberg & Claffey, 1996; Henry & Fraser-Reid, 1995; Timmers *et al.*, 1996). The Simmons-Smith cyclopropanation involves treating a glycal with CH_2_I_2_/Zn/CuCl activated with acetyl chloride. Under those conditions a cyclopropane *syn* to the oxygen of the C-3 of the respective glycal is formed. On the other hand, the dihalocarbene cyclopropanation of glycals affords a cyclopropane with a stereochemistry opposite to that of the Simmons-Smith cyclopropanation. Herein we report the dihalocarbene cyclopropanation of per-*p*-methoxybenzyl protected glucal (A in Fig. 2) and the confirmation of the stereochemistry of the cyclopropanated product (I in Fig. 2).

The title compound (see Fig. 1 and Scheme 1) crystallizes in the *P*2_1_ (*Z*=2) space group resulting in molecules lying on general positions in the unit cell. All bond lengths are within their normal ranges (Allen *et al.*, 1987). The pyran ring is in a twist-boat conformation with ring puckering parameters of q_2_ = 0.7035 (14) Å, q_3_ = -0.0851 (15) Å, Q = 0.7086 (14)Å and φ_2_ = 346.57 (12)° (see Cremer & Pople, 1975). The *O-p*-methoxybenzyl groups are all in equatorial positions with the methoxy groups nearly coplanar with their respective rings (dihedral angles of 0.16 (27)° and 9.36 (21)° for rings C9—C14 and C17—C22 respectively). The aromatic rings adopt orientations enabling them to participate in C—H···*Cg* interactions with neighboring methoxy groups (Table 1).  There are also several weak C—H···O interactions (Table 1) that aid in the stabilization of the crystal structure.

### Experimental

50% aq NaOH (3 mL) was added to a vigorously stirred solution of glucal (see A in Fig. 2; 0.5 g, 0.99 mmol) in chloroform (5 ml) containing benzyltriethylammonium chloride (0.11 g, 0.49 mmol). After stirring at 35°C overnight, the reaction mixture was diluted with water and the aqueous phase was extracted with dichloromethane. The combined organic phases were dried over MgSO_4_, filtered and evaporated *in vacuo*. Chromatography on silica gel (ethyl acetate/hexane, 5:95) of the residue and recrystallization from hexane gave the title compound in 80% yield as a white solid.

Analytical data: ^1^H NMR (CDCl_3_, 400 MHz) δ 7.32 (d, *J* = 8.0 Hz, 2H), 7.21 (d, *J* = 8.4 Hz, 2H), 7.11 (d, *J* = 8.0 Hz, 2H), 6.88 (d, *J* = 8.4 Hz, 2H), 6.86 (d, *J* = 8.0 Hz, 2H), 6.81 (d, *J* = 8.0 Hz, 2H), 4.77 (d, *J* = 10.8 Hz, 2H), 4.70 (d, *J* = 11.2 Hz, 2H), 4.60 (d, *J* = 11.2 Hz, 2H), 4.46 (d, *J* = 12.4 Hz, 2H), 4.42 (d, *J* = 12.4 Hz, 2H), 4.35 (d, *J* = 11.6 Hz, 2H), 3.84 (d, *J* = 8.0 Hz, 1H), 3.82–3.60 (m, 12H), 3.58–3.40 (m, 2H), 1.73 (dd, *J* = 4.2 and 7.8 Hz, 1H); ^13^C NMR (CDCl_3_, 100 MHz) δ 159.4, 159.3, 159.2, 130.4, 130.0, 129.8, 129.7, 129.5, 129.3, 113.9, 113.8, 113.7, 79.8, 74.7, 74.1, 72.9, 71.5, 69.7, 61.5, 58.9, 55.2, 34.3.

### Refinement

All hydrogen atoms were positioned in geometrically idealized positions with C—H = 1.00 Å, 0.99 Å, 0.98 Å and 0.95 Å for methine, methylene, methyl and aromatic H atoms respectively. All hydrogen atoms were allowed to ride on their parent atoms with *U*_iso_(H) = 1.2*U*_eq_, except for methyl where *U*_iso_(H) = 1.5*U*_eq_ was utilized. The initial positions of methyl hydrogen atoms were located from a Fourier difference map and refined as fixed rotor. The Friedel pair coverage for the collection is fairly low, possibly due to an inadequate collection strategy. A recollection was not deemed neccesary since the D-enantiomer can be unambiguously assigned from the known configuration of the starting glucal. The Flack parameter refined to 0.03 (3). The highest residual electron density of 0.28 e.Å^-3^ is 0.77 Å from Cl2 and has no physical meaning.

### Crystal data

**Table:**

C_31_H_34_Cl_2_O_7_	*F*(000) = 620
*M**_r_* = 589.48	*D*_x_ = 1.364 Mg m^−^^3^
Monoclinic, *P*2_1_	Melting point = 343–345 K
Hall symbol: P 2yb	Mo *K*α radiation, λ = 0.71073 Å
*a* = 5.3480 (4) Å	Cell parameters from 9926 reflections
*b* = 18.1110 (14) Å	θ = 2.6–28.3°
*c* = 14.8230 (11) Å	µ = 0.27 mm^−^^1^
β = 91.162 (2)°	*T* = 100 K
*V* = 1435.43 (19) Å^3^	Cuboid, colourless
*Z* = 2	0.53 × 0.44 × 0.39 mm

### Data collection

**Table:**

Bruker KappaCCD APEX DUO 4K diffractometer	4635 reflections with *I* > 2σ(*I*)
graphite	*R*_int_ = 0.026
φ and ω scans	θ_max_ = 28.3°, θ_min_ = 1.8°
Absorption correction: multi-scan (*SADABS*; Bruker, 2007)\bbr00	*h* = −7→7
*T*_min_ = 0.869, *T*_max_ = 0.901	*k* = −11→24
13864 measured reflections	*l* = −19→19
4689 independent reflections	

### Refinement

**Table:**

Refinement on *F*^2^	Secondary atom site location: difference Fourier map
Least-squares matrix: full	Hydrogen site location: inferred from neighbouring sites
*R*[*F*^2^ > 2σ(*F*^2^)] = 0.023	H-atom parameters constrained
*wR*(*F*^2^) = 0.062	*w* = 1/[σ^2^(*F*_o_^2^) + (0.0333*P*)^2^ + 0.3097*P*] where *P* = (*F*_o_^2^ + 2*F*_c_^2^)/3
*S* = 1.04	(Δ/σ)_max_ = 0.001
4689 reflections	Δρ_max_ = 0.28 e Å^−^^3^
364 parameters	Δρ_min_ = −0.16 e Å^−^^3^
1 restraint	Absolute structure: Flack (1983), 1023 Friedel pairs
Primary atom site location: structure-invariant direct methods	Flack parameter: 0.03 (3)

### Special details

**Table:**

Experimental. The intensity data was collected on a Bruker *APEX* Duo 4 K KappaCCD diffractometer using an exposure time of 60 s/frame. A total of 1324 frames were collected with a frame width of 0.5° covering up to θ = 28.3° with 99.6% completeness accomplished.
Geometry. All e.s.d.'s (except the e.s.d. in the dihedral angle between two l.s. planes) are estimated using the full covariance matrix. The cell e.s.d.'s are taken into account individually in the estimation of e.s.d.'s in distances, angles and torsion angles; correlations between e.s.d.'s in cell parameters are only used when they are defined by crystal symmetry. An approximate (isotropic) treatment of cell e.s.d.'s is used for estimating e.s.d.'s involving l.s. planes.
Refinement. Refinement of *F*^2^ against ALL reflections. The weighted *R*-factor *wR* and goodness of fit *S* are based on *F*^2^, conventional *R*-factors *R* are based on *F*, with *F* set to zero for negative *F*^2^. The threshold expression of *F*^2^ > σ(*F*^2^) is used only for calculating *R*-factors(gt) *etc*. and is not relevant to the choice of reflections for refinement. *R*-factors based on *F*^2^ are statistically about twice as large as those based on *F*, and *R*- factors based on ALL data will be even larger.

### Fractional atomic coordinates and isotropic or equivalent isotropic displacement parameters (Å^2^)

**Table:**

	*x*	*y*	*z*	*U*_iso_*/*U*_eq_	
C1	0.5746 (3)	0.37183 (8)	0.12955 (9)	0.0159 (3)	
H1	0.7267	0.4023	0.1182	0.019*	
C2	0.3481 (3)	0.41817 (8)	0.09697 (9)	0.0141 (3)	
H2	0.2059	0.4114	0.139	0.017*	
C3	0.2681 (2)	0.39508 (8)	0.00162 (9)	0.0135 (2)	
H3	0.4112	0.401	−0.0402	0.016*	
C4	0.1924 (2)	0.31426 (8)	0.00670 (9)	0.0143 (2)	
H4	0.0124	0.3039	0.0186	0.017*	
C5	0.3834 (2)	0.26695 (8)	0.05720 (9)	0.0162 (3)	
H5	0.3203	0.2289	0.1001	0.019*	
C6	0.3340 (3)	0.25520 (8)	−0.04201 (9)	0.0160 (3)	
C7	0.5711 (3)	0.35798 (9)	0.23087 (9)	0.0203 (3)	
H7A	0.7335	0.3372	0.2517	0.024*	
H7B	0.5436	0.405	0.2632	0.024*	
C8	0.2633 (3)	0.31871 (11)	0.33538 (10)	0.0256 (3)	
H8A	0.1314	0.2811	0.3435	0.031*	
H8B	0.1815	0.3677	0.3354	0.031*	
C9	0.4444 (3)	0.31468 (10)	0.41480 (9)	0.0225 (3)	
C10	0.6119 (3)	0.25666 (10)	0.42635 (10)	0.0252 (3)	
H10	0.6156	0.2183	0.3826	0.03*	
C11	0.7754 (3)	0.25366 (10)	0.50125 (10)	0.0263 (3)	
H11	0.8919	0.2143	0.5075	0.032*	
C12	0.7657 (3)	0.30875 (11)	0.56633 (11)	0.0286 (3)	
C13	0.5962 (4)	0.36662 (12)	0.55592 (13)	0.0366 (4)	
H13	0.5882	0.4041	0.6006	0.044*	
C14	0.4393 (4)	0.36961 (11)	0.48059 (12)	0.0315 (4)	
H14	0.3263	0.4098	0.4736	0.038*	
C15	1.0910 (4)	0.25312 (14)	0.65659 (15)	0.0446 (5)	
H15A	1.0016	0.2063	0.6636	0.067*	
H15B	1.1919	0.263	0.7112	0.067*	
H15C	1.2005	0.25	0.6045	0.067*	
C16	0.2468 (3)	0.54661 (9)	0.12345 (9)	0.0173 (3)	
H16A	0.0832	0.5318	0.0967	0.021*	
H16B	0.2925	0.5951	0.0978	0.021*	
C17	0.2251 (3)	0.55341 (8)	0.22438 (9)	0.0167 (3)	
C18	0.0275 (3)	0.52223 (9)	0.27015 (10)	0.0207 (3)	
H18	−0.1031	0.4985	0.2368	0.025*	
C19	0.0161 (3)	0.52497 (10)	0.36452 (10)	0.0228 (3)	
H19	−0.12	0.5032	0.395	0.027*	
C20	0.2065 (3)	0.55998 (9)	0.41261 (10)	0.0206 (3)	
C21	0.4042 (3)	0.59335 (10)	0.36741 (10)	0.0229 (3)	
H21	0.5321	0.6185	0.4006	0.027*	
C22	0.4127 (3)	0.58960 (9)	0.27443 (10)	0.0206 (3)	
H22	0.5479	0.6119	0.244	0.025*	
C23	0.0028 (4)	0.54396 (14)	0.55311 (11)	0.0376 (5)	
H23A	−0.145	0.5696	0.5286	0.056*	
H23B	0.0258	0.5565	0.6171	0.056*	
H23C	−0.0205	0.4905	0.5469	0.056*	
C24	0.0170 (3)	0.43201 (9)	−0.12203 (9)	0.0156 (3)	
H24A	−0.0304	0.3803	−0.1355	0.019*	
H24B	0.1702	0.4437	−0.1556	0.019*	
C25	−0.1908 (3)	0.48286 (8)	−0.15179 (8)	0.0149 (3)	
C26	−0.3255 (3)	0.52635 (9)	−0.09302 (9)	0.0157 (3)	
H26	−0.2869	0.5243	−0.0302	0.019*	
C27	−0.5166 (3)	0.57303 (9)	−0.12405 (9)	0.0165 (3)	
H27	−0.608	0.6019	−0.0826	0.02*	
C28	−0.5722 (3)	0.57693 (9)	−0.21629 (9)	0.0174 (3)	
C29	−0.4381 (3)	0.53311 (10)	−0.27614 (9)	0.0223 (3)	
H29	−0.4757	0.5352	−0.339	0.027*	
C30	−0.2511 (3)	0.48674 (9)	−0.24411 (9)	0.0202 (3)	
H30	−0.1619	0.457	−0.2854	0.024*	
C31	−0.9079 (3)	0.66226 (9)	−0.19589 (10)	0.0229 (3)	
H31A	−0.9814	0.6293	−0.1512	0.034*	
H31B	−1.0416	0.6865	−0.231	0.034*	
H31C	−0.8057	0.6998	−0.1649	0.034*	
O1	0.60536 (18)	0.30368 (6)	0.08006 (7)	0.0172 (2)	
O2	0.3745 (2)	0.30744 (7)	0.24945 (7)	0.0234 (2)	
O3	0.43372 (19)	0.49265 (6)	0.10078 (7)	0.0165 (2)	
O4	0.06550 (18)	0.44011 (6)	−0.02738 (6)	0.0157 (2)	
O5	0.9144 (3)	0.31154 (10)	0.64279 (9)	0.0426 (4)	
O6	0.2183 (2)	0.56619 (8)	0.50476 (7)	0.0269 (3)	
O7	−0.7547 (2)	0.62045 (7)	−0.25488 (7)	0.0228 (2)	
Cl1	0.55865 (6)	0.27721 (2)	−0.12181 (2)	0.02147 (8)	
Cl2	0.16666 (7)	0.17496 (2)	−0.07142 (3)	0.02262 (8)	

### Atomic displacement parameters (Å^2^)

**Table:**

	*U*^11^	*U*^22^	*U*^33^	*U*^12^	*U*^13^	*U*^23^
C1	0.0144 (6)	0.0158 (7)	0.0173 (6)	0.0011 (5)	−0.0020 (5)	−0.0001 (5)
C2	0.0148 (6)	0.0141 (7)	0.0136 (5)	−0.0005 (5)	0.0001 (4)	0.0008 (5)
C3	0.0134 (6)	0.0132 (6)	0.0138 (5)	0.0018 (5)	−0.0003 (4)	0.0003 (5)
C4	0.0131 (6)	0.0128 (6)	0.0170 (5)	−0.0003 (5)	−0.0001 (4)	0.0000 (5)
C5	0.0153 (6)	0.0140 (7)	0.0192 (6)	0.0003 (5)	−0.0019 (5)	0.0014 (5)
C6	0.0135 (6)	0.0146 (7)	0.0198 (6)	−0.0024 (5)	−0.0001 (5)	−0.0021 (5)
C7	0.0213 (7)	0.0225 (8)	0.0170 (6)	−0.0019 (6)	−0.0056 (5)	0.0017 (6)
C8	0.0262 (7)	0.0330 (9)	0.0175 (6)	−0.0021 (7)	−0.0026 (5)	0.0029 (6)
C9	0.0256 (7)	0.0240 (8)	0.0177 (6)	−0.0037 (6)	−0.0018 (5)	0.0032 (6)
C10	0.0315 (8)	0.0255 (9)	0.0186 (6)	−0.0003 (7)	−0.0004 (6)	−0.0021 (6)
C11	0.0296 (8)	0.0246 (8)	0.0244 (7)	0.0027 (7)	−0.0017 (6)	0.0036 (6)
C12	0.0313 (8)	0.0304 (9)	0.0238 (7)	−0.0019 (7)	−0.0094 (6)	0.0003 (7)
C13	0.0479 (11)	0.0282 (10)	0.0332 (9)	0.0030 (9)	−0.0153 (8)	−0.0117 (8)
C14	0.0377 (9)	0.0235 (9)	0.0329 (8)	0.0042 (7)	−0.0107 (7)	−0.0032 (7)
C15	0.0461 (11)	0.0433 (12)	0.0434 (10)	0.0023 (10)	−0.0240 (9)	0.0071 (9)
C16	0.0207 (6)	0.0159 (7)	0.0154 (6)	0.0036 (6)	−0.0008 (5)	−0.0011 (5)
C17	0.0185 (6)	0.0156 (7)	0.0160 (6)	0.0042 (6)	−0.0008 (5)	−0.0022 (5)
C18	0.0200 (6)	0.0222 (8)	0.0198 (6)	−0.0007 (6)	0.0000 (5)	−0.0044 (6)
C19	0.0225 (7)	0.0252 (8)	0.0210 (6)	−0.0023 (6)	0.0051 (5)	−0.0024 (6)
C20	0.0226 (7)	0.0228 (8)	0.0164 (6)	0.0038 (6)	0.0000 (5)	−0.0013 (6)
C21	0.0209 (7)	0.0283 (9)	0.0193 (6)	−0.0014 (6)	−0.0033 (5)	−0.0036 (6)
C22	0.0193 (7)	0.0232 (8)	0.0194 (6)	−0.0020 (6)	0.0014 (5)	−0.0017 (6)
C23	0.0373 (9)	0.0563 (14)	0.0193 (7)	−0.0073 (10)	0.0069 (7)	−0.0003 (8)
C24	0.0193 (6)	0.0150 (7)	0.0124 (5)	0.0003 (5)	−0.0012 (4)	−0.0006 (5)
C25	0.0164 (6)	0.0144 (6)	0.0140 (5)	−0.0032 (5)	−0.0007 (5)	0.0012 (5)
C26	0.0186 (6)	0.0159 (7)	0.0125 (5)	−0.0029 (5)	−0.0008 (5)	0.0014 (5)
C27	0.0185 (6)	0.0151 (7)	0.0160 (6)	−0.0022 (5)	0.0016 (5)	−0.0004 (5)
C28	0.0171 (6)	0.0177 (7)	0.0173 (6)	−0.0008 (5)	−0.0005 (5)	0.0028 (5)
C29	0.0247 (7)	0.0285 (9)	0.0134 (5)	0.0037 (7)	−0.0020 (5)	−0.0002 (6)
C30	0.0238 (7)	0.0234 (8)	0.0134 (6)	0.0036 (6)	−0.0005 (5)	−0.0025 (6)
C31	0.0211 (7)	0.0199 (8)	0.0277 (7)	0.0014 (6)	−0.0016 (6)	−0.0004 (6)
O1	0.0137 (4)	0.0163 (5)	0.0215 (4)	0.0012 (4)	−0.0024 (3)	−0.0021 (4)
O2	0.0285 (5)	0.0264 (6)	0.0151 (4)	−0.0079 (5)	−0.0027 (4)	0.0027 (4)
O3	0.0182 (5)	0.0140 (5)	0.0176 (4)	−0.0005 (4)	0.0023 (4)	−0.0028 (4)
O4	0.0185 (5)	0.0161 (5)	0.0123 (4)	0.0046 (4)	−0.0021 (3)	0.0003 (4)
O5	0.0490 (8)	0.0447 (9)	0.0331 (6)	0.0073 (7)	−0.0226 (6)	−0.0062 (6)
O6	0.0290 (6)	0.0368 (7)	0.0149 (5)	−0.0019 (5)	0.0007 (4)	−0.0011 (5)
O7	0.0223 (5)	0.0268 (6)	0.0192 (5)	0.0066 (5)	−0.0027 (4)	0.0030 (4)
Cl1	0.01893 (15)	0.02378 (18)	0.02188 (15)	−0.00135 (14)	0.00472 (12)	−0.00492 (14)
Cl2	0.02241 (16)	0.01435 (16)	0.03092 (17)	−0.00321 (14)	−0.00394 (13)	−0.00353 (14)

### Geometric parameters (Å, °)

**Table:**

C1—O1	1.4469 (18)	C15—H15C	0.98
C1—C7	1.5231 (18)	C16—O3	1.4425 (17)
C1—C2	1.5433 (19)	C16—C17	1.5080 (18)
C1—H1	1	C16—H16A	0.99
C2—O3	1.4252 (18)	C16—H16B	0.99
C2—C3	1.5267 (18)	C17—C18	1.388 (2)
C2—H2	1	C17—C22	1.399 (2)
C3—O4	1.4159 (16)	C18—C19	1.402 (2)
C3—C4	1.521 (2)	C18—H18	0.95
C3—H3	1	C19—C20	1.385 (2)
C4—C6	1.504 (2)	C19—H19	0.95
C4—C5	1.5189 (19)	C20—O6	1.3707 (17)
C4—H4	1	C20—C21	1.400 (2)
C5—O1	1.3966 (17)	C21—C22	1.382 (2)
C5—C6	1.5039 (19)	C21—H21	0.95
C5—H5	1	C22—H22	0.95
C6—Cl1	1.7489 (14)	C23—O6	1.427 (2)
C6—Cl2	1.7570 (15)	C23—H23A	0.98
C7—O2	1.4252 (19)	C23—H23B	0.98
C7—H7A	0.99	C23—H23C	0.98
C7—H7B	0.99	C24—O4	1.4292 (15)
C8—O2	1.4312 (18)	C24—C25	1.503 (2)
C8—C9	1.511 (2)	C24—H24A	0.99
C8—H8A	0.99	C24—H24B	0.99
C8—H8B	0.99	C25—C26	1.3869 (19)
C9—C10	1.389 (2)	C25—C30	1.4013 (18)
C9—C14	1.394 (2)	C26—C27	1.397 (2)
C10—C11	1.400 (2)	C26—H26	0.95
C10—H10	0.95	C27—C28	1.3951 (18)
C11—C12	1.389 (2)	C27—H27	0.95
C11—H11	0.95	C28—O7	1.3705 (18)
C12—O5	1.3724 (19)	C28—C29	1.399 (2)
C12—C13	1.392 (3)	C29—C30	1.383 (2)
C13—C14	1.384 (2)	C29—H29	0.95
C13—H13	0.95	C30—H30	0.95
C14—H14	0.95	C31—O7	1.4277 (18)
C15—O5	1.430 (3)	C31—H31A	0.98
C15—H15A	0.98	C31—H31B	0.98
C15—H15B	0.98	C31—H31C	0.98
			
O1—C1—C7	111.30 (12)	H15A—C15—H15C	109.5
O1—C1—C2	113.69 (11)	H15B—C15—H15C	109.5
C7—C1—C2	111.89 (11)	O3—C16—C17	110.74 (11)
O1—C1—H1	106.5	O3—C16—H16A	109.5
C7—C1—H1	106.5	C17—C16—H16A	109.5
C2—C1—H1	106.5	O3—C16—H16B	109.5
O3—C2—C3	112.32 (11)	C17—C16—H16B	109.5
O3—C2—C1	104.64 (11)	H16A—C16—H16B	108.1
C3—C2—C1	110.13 (11)	C18—C17—C22	118.47 (13)
O3—C2—H2	109.9	C18—C17—C16	121.78 (13)
C3—C2—H2	109.9	C22—C17—C16	119.71 (13)
C1—C2—H2	109.9	C17—C18—C19	121.48 (14)
O4—C3—C4	111.51 (11)	C17—C18—H18	119.3
O4—C3—C2	108.76 (11)	C19—C18—H18	119.3
C4—C3—C2	106.72 (11)	C20—C19—C18	118.90 (14)
O4—C3—H3	109.9	C20—C19—H19	120.5
C4—C3—H3	109.9	C18—C19—H19	120.5
C2—C3—H3	109.9	O6—C20—C19	124.68 (14)
C6—C4—C5	59.67 (9)	O6—C20—C21	114.93 (13)
C6—C4—C3	121.57 (11)	C19—C20—C21	120.39 (13)
C5—C4—C3	112.98 (11)	C22—C21—C20	119.77 (14)
C6—C4—H4	116.6	C22—C21—H21	120.1
C5—C4—H4	116.6	C20—C21—H21	120.1
C3—C4—H4	116.6	C21—C22—C17	120.96 (14)
O1—C5—C6	115.86 (11)	C21—C22—H22	119.5
O1—C5—C4	114.25 (12)	C17—C22—H22	119.5
C6—C5—C4	59.67 (9)	O6—C23—H23A	109.5
O1—C5—H5	118	O6—C23—H23B	109.5
C6—C5—H5	118	H23A—C23—H23B	109.5
C4—C5—H5	118	O6—C23—H23C	109.5
C5—C6—C4	60.66 (9)	H23A—C23—H23C	109.5
C5—C6—Cl1	121.32 (10)	H23B—C23—H23C	109.5
C4—C6—Cl1	121.48 (11)	O4—C24—C25	110.10 (11)
C5—C6—Cl2	115.98 (10)	O4—C24—H24A	109.6
C4—C6—Cl2	116.64 (10)	C25—C24—H24A	109.6
Cl1—C6—Cl2	111.97 (8)	O4—C24—H24B	109.6
O2—C7—C1	108.68 (11)	C25—C24—H24B	109.6
O2—C7—H7A	110	H24A—C24—H24B	108.2
C1—C7—H7A	110	C26—C25—C30	118.22 (13)
O2—C7—H7B	110	C26—C25—C24	123.65 (12)
C1—C7—H7B	110	C30—C25—C24	118.13 (12)
H7A—C7—H7B	108.3	C25—C26—C27	121.51 (12)
O2—C8—C9	114.52 (13)	C25—C26—H26	119.2
O2—C8—H8A	108.6	C27—C26—H26	119.2
C9—C8—H8A	108.6	C28—C27—C26	119.56 (13)
O2—C8—H8B	108.6	C28—C27—H27	120.2
C9—C8—H8B	108.6	C26—C27—H27	120.2
H8A—C8—H8B	107.6	O7—C28—C27	125.03 (13)
C10—C9—C14	118.39 (15)	O7—C28—C29	115.55 (12)
C10—C9—C8	122.28 (15)	C27—C28—C29	119.41 (14)
C14—C9—C8	119.29 (16)	C30—C29—C28	120.22 (13)
C9—C10—C11	121.18 (16)	C30—C29—H29	119.9
C9—C10—H10	119.4	C28—C29—H29	119.9
C11—C10—H10	119.4	C29—C30—C25	121.08 (13)
C12—C11—C10	119.42 (16)	C29—C30—H30	119.5
C12—C11—H11	120.3	C25—C30—H30	119.5
C10—C11—H11	120.3	O7—C31—H31A	109.5
O5—C12—C11	124.79 (17)	O7—C31—H31B	109.5
O5—C12—C13	115.41 (16)	H31A—C31—H31B	109.5
C11—C12—C13	119.80 (15)	O7—C31—H31C	109.5
C14—C13—C12	120.10 (17)	H31A—C31—H31C	109.5
C14—C13—H13	119.9	H31B—C31—H31C	109.5
C12—C13—H13	119.9	C5—O1—C1	115.13 (10)
C13—C14—C9	121.09 (17)	C7—O2—C8	113.70 (12)
C13—C14—H14	119.5	C2—O3—C16	115.26 (10)
C9—C14—H14	119.5	C3—O4—C24	111.19 (10)
O5—C15—H15A	109.5	C12—O5—C15	117.45 (16)
O5—C15—H15B	109.5	C20—O6—C23	117.13 (13)
H15A—C15—H15B	109.5	C28—O7—C31	117.52 (11)
O5—C15—H15C	109.5		
			
O1—C1—C2—O3	−140.75 (11)	C22—C17—C18—C19	−1.4 (2)
C7—C1—C2—O3	92.10 (13)	C16—C17—C18—C19	176.15 (15)
O1—C1—C2—C3	−19.84 (16)	C17—C18—C19—C20	0.3 (3)
C7—C1—C2—C3	−146.98 (12)	C18—C19—C20—O6	−179.70 (15)
O3—C2—C3—O4	−61.95 (14)	C18—C19—C20—C21	1.3 (3)
C1—C2—C3—O4	−178.14 (11)	O6—C20—C21—C22	179.18 (15)
O3—C2—C3—C4	177.64 (10)	C19—C20—C21—C22	−1.7 (3)
C1—C2—C3—C4	61.44 (13)	C20—C21—C22—C17	0.6 (3)
O4—C3—C4—C6	125.64 (12)	C18—C17—C22—C21	1.0 (2)
C2—C3—C4—C6	−115.73 (13)	C16—C17—C22—C21	−176.63 (15)
O4—C3—C4—C5	−166.90 (10)	O4—C24—C25—C26	−4.7 (2)
C2—C3—C4—C5	−48.27 (14)	O4—C24—C25—C30	175.07 (13)
C6—C4—C5—O1	106.90 (13)	C30—C25—C26—C27	0.0 (2)
C3—C4—C5—O1	−7.35 (15)	C24—C25—C26—C27	179.74 (14)
C3—C4—C5—C6	−114.25 (12)	C25—C26—C27—C28	−0.8 (2)
O1—C5—C6—C4	−104.19 (14)	C26—C27—C28—O7	179.99 (14)
O1—C5—C6—Cl1	6.86 (18)	C26—C27—C28—C29	1.0 (2)
C4—C5—C6—Cl1	111.05 (13)	O7—C28—C29—C30	−179.52 (15)
O1—C5—C6—Cl2	148.46 (10)	C27—C28—C29—C30	−0.4 (2)
C4—C5—C6—Cl2	−107.35 (11)	C28—C29—C30—C25	−0.4 (3)
C3—C4—C6—C5	99.86 (14)	C26—C25—C30—C29	0.6 (2)
C5—C4—C6—Cl1	−110.80 (12)	C24—C25—C30—C29	−179.18 (15)
C3—C4—C6—Cl1	−10.94 (18)	C6—C5—O1—C1	120.36 (13)
C5—C4—C6—Cl2	106.27 (12)	C4—C5—O1—C1	53.75 (15)
C3—C4—C6—Cl2	−153.86 (10)	C7—C1—O1—C5	88.59 (14)
O1—C1—C7—O2	−57.49 (15)	C2—C1—O1—C5	−38.87 (15)
C2—C1—C7—O2	70.93 (16)	C1—C7—O2—C8	−148.42 (13)
O2—C8—C9—C10	−49.6 (2)	C9—C8—O2—C7	−57.37 (19)
O2—C8—C9—C14	132.47 (17)	C3—C2—O3—C16	93.89 (13)
C14—C9—C10—C11	−1.1 (2)	C1—C2—O3—C16	−146.66 (10)
C8—C9—C10—C11	−179.03 (15)	C17—C16—O3—C2	85.55 (14)
C9—C10—C11—C12	1.6 (3)	C4—C3—O4—C24	−74.90 (13)
C10—C11—C12—O5	179.50 (17)	C2—C3—O4—C24	167.69 (11)
C10—C11—C12—C13	−0.7 (3)	C25—C24—O4—C3	−177.44 (11)
O5—C12—C13—C14	179.22 (19)	C11—C12—O5—C15	−0.4 (3)
C11—C12—C13—C14	−0.6 (3)	C13—C12—O5—C15	179.80 (19)
C12—C13—C14—C9	1.1 (3)	C19—C20—O6—C23	−9.0 (3)
C10—C9—C14—C13	−0.2 (3)	C21—C20—O6—C23	170.06 (17)
C8—C9—C14—C13	177.76 (18)	C27—C28—O7—C31	−3.3 (2)
O3—C16—C17—C18	−103.82 (16)	C29—C28—O7—C31	175.76 (14)
O3—C16—C17—C22	73.73 (18)		

### Hydrogen-bond geometry (Å, °)

**Table:**

Cg1, Cg2 and Cg3 are the centroids of the C17–C22, C9–C14 and C25–C30 rings, respectively.

**Table:**

*D*—H···*A*	*D*—H	H···*A*	*D*···*A*	*D*—H···*A*
C4—H4···O1^i^	1.00	2.38	3.3487 (16)	164
C23—H23B···O7^ii^	0.98	2.50	3.399 (2)	153
C26—H26···O3^i^	0.95	2.54	3.2301 (16)	130
C31—H31C···O1^iii^	0.98	2.49	3.466 (2)	172
C15—H15A···Cg1^iv^	0.98	2.95	3.927 (3)	174
C15—H15C···Cg2^v^	0.98	2.99	3.873 (2)	151
C24—H24B···Cg3^v^	0.99	2.90	3.7983 (17)	152

Symmetry codes: (i) *x*−1, *y*, *z*; (ii) *x*+1, *y*, *z*+1; (iii) −*x*, *y*+1/2, −*z*; (iv) −*x*+1, *y*−1/2, −*z*+1; (v) *x*+1, *y*, *z*.
